# Pea protein hydrolysate reduces blood glucose in high-fat diet and streptozotocin-induced diabetic mice

**DOI:** 10.3389/fnut.2023.1298046

**Published:** 2023-12-12

**Authors:** Wang Liao, Xinyi Cao, Hui Xia, Shaokang Wang, Liang Chen, Guiju Sun

**Affiliations:** ^1^Key Laboratory of Environmental Medicine and Engineering of Ministry of Education, Department of Nutrition and Food Hygiene, School of Public Health, Southeast University, Nanjing, China; ^2^Public Service Platform of South China Sea for R&D Marine Biomedicine Resources, The Marine Biomedical Research Institute, Guangdong Medical University, Zhanjiang, China

**Keywords:** pea protein hydrolysate, type 2 diabetes, gluconeogenesis, insulin sensitivity, chronic disease

## Abstract

**Introduction:**

Food proteins have been recognized as an ideal source to release bioactive peptides with the potential to intervene nutrition related chronic diseases, such as cardiovascular diseases, obesity and diabetes. Our previous studies showed that pea protein hydrolysate (PPH) could suppress hepatic glucose production in hepatic cells via inhibiting the gluconeogenic signaling. Thus, we hypothesized that PPH could play the hypoglycemic role in vivo.

**Methods:**

In the present study, the mice model with type 2 diabetic mellitus (T2DM) was developed by high-fat diet and low dose of streptozotocin injections. PPH was administered orally with a dosage of 1000 mg/kg body weight for 9 weeks, followed by the downstream biomedical analyses.

**Results:**

The results showed that the 9-week treatment of PPH could reduce fasting blood glucose by 29.6% and improve glucose tolerance in the T2DM mice. The associated mechanisms included suppression of the gluconeogenic pathway, activation of the insulin signaling and modulation of the renin angiotensin system in the liver of the diabetic mice. In addition, the levels of pro-inflammatory markers in both liver and serum were reduced by the PPH treatment.

**Conclusion:**

The hypoglycemic effect of PPH in T2DM mice was demonstrated in the present study. Findings from this study could provide rationale to incorporate PPH into functional foods or nutraceuticals for glycemic control.

## 1 Introduction

Diabetes mellitus (DM) has been considered as one of the most serious chronic diseases in the world, which is associated with complex complications, disability, and reduced life expectancy (1). As estimated, global DM-related health expenditures had reached 966 USD in 2021 and was projected to 1,054 USD by 2045. Approximately, T2DM accounts for 90% of all the DM cases (2). In terms of the pathophysiology, the unbalanced diet, such as high-caloric western diet, could result in dyslipidemia and increase oxidative stress, which activates a number of proinflammatory pathways that consequently lead to insulin resistance and T2DM (3). On the other hand, the insulin resistance under the fasting status could trigger increased hepatic glucose output, which is another essential factor of the T2DM etiology (4). Thus, improvement of the insulin sensitivity or/and suppression of hepatic gluconeogenesis has been considered as a key strategy for the management of T2DM.

Due to the side effects of hypoglycemic drugs, naturally derived bioactive compounds have gained great attractions for the intervention of T2DM. Plant-based products have emerged as an important food source, which is owing to their green nature and health benefits (5). As a representative of plant-based food source, legume is a stable type of food involved in the diet of diverse cultures (6). Of note, there is growing evidence showing the health benefits of a high legume consumption. It was reported that daily dietary inclusion of 150 g of cooked legumes could contribute to lowering cardiovascular diseases-related mortality (7). Thereby, legumes have been an interesting source for the identification of bioactive compounds due to the low cost and high nutritional value. A wide range of bioactivities of the legume derived phytochemicals and bioactive peptides have been documented, which showed a great potential of these compound in the intervention of nutrition related chronic diseases, such as obesity, cardiovascular diseases and T2DM (6, 8, 9).

Pea (*Pisum sativum*) is one of the major legumes in the world with high protein content and good availability (10). Notably, the anti-diabetic activity of pea protein-derived peptides have been suggested, which is contributed by inhibiting the activity of α-glucosidase or dipeptidyl peptidase 4 (DPP-4) (8). However, the *in vivo* hypoglycemic effect and the underlying molecular mechanisms of pea peptides are ambiguous. Our recent study profiled the peptide composition of pea protein hydrolysate (PPH). More importantly, we found that PPH could suppress gluconeogenic pathway and inhibit glucose production in hepatic cells (11), which indicates the role of PPH in regulating signaling pathways to sustain glucose homeostasis.

To further investigate the *in vivo* hypoglycemic effect of PPH and elucidate the mechanistic actions, the present study aims at investigating the hypoglycemic effect and mechanisms of PPH in a T2DM mouse model established by high-fat diet and low dose of streptozotocin (STZ) injections.

## 2 Methods and materials

### 2.1 Mouse model

All animal procedures were approved by the Animal Care and Use Committee of the Southeast University (Approval #20210221003). The T2DM mouse model could be established by the high-fat-diet and low dose of STZ injection (12), which has been used in a number of studies evaluating the bioactivity of natural compounds (13–15). Male C57BL/6J mice (5 weeks-old) were purchased from Huachuang Sino Pharmatech Co., Ltd. (Taizhou, Jiangsu, China) and maintained in the animal facility for 1 week. The animals were assigned into 3 groups randomly. Based on the power analysis and successful rate of the T2DM model establishment, 6 animals were assigned to each group. The normal control group was kept with the normal chow diet (10 kcal% of fat, 70 kcal% of carbohydrate and 20 kcal% of protein, Xietong Organism, Nanjing, China). The T2DM group and the PPH group were fed by the high-fat-diet (60 kcal% of fat, 20 kcal% of carbohydrate and 20 kcal% of protein, Xietong Organism) for 4 weeks. Detailed diet compositions of the high-fat-diet was shown in Table 1. In the fifth week, the animals in the T2DM and the PPH group were injected with 40 mg/kg of STZ (Solarbio Science & Technology Co., Ltd., Beijing, China) dissolved in the citrate buffer for consecutive 5 days. Meanwhile, the animals in the control group were injected by the citrate buffer. After 2 weeks of the injection, fasting blood glucose (after food deprivation for 12 h) was measured. Animals (injected with STZ) with blood glucose higher than 11.1 mM were used for the following experiments.

**Table 1:** Ingredient composition of the high-fat diet (60% fat kcal) diet fed to mice.

Ingredient	Normal chow diet (g/kg)	High-fat (60% fat kcal) diet (g/kg)
Casein	189.56	258.45
L-cystine	2.84	3.88
Carbohydrate (corn starch)	479.79	—
Carbohydrate (Lodex 10)	118.48	161.53
Carbohydrate (sucrose)	69.00	94.08
Fiber	47.39	64.61
Soybean oil	23.70	32.31
Lard	18.96	316.60
Mineral mix	47.39	64.61
Vitamin mix	0.95	1.29
Choline bitartrate	1.90	2.58
FD&C Blue Dye #1	0.01	0.06
FD&C Yellow Dye #5	0.04	—

### 2.2 Animal treatment

**Figure 1:**
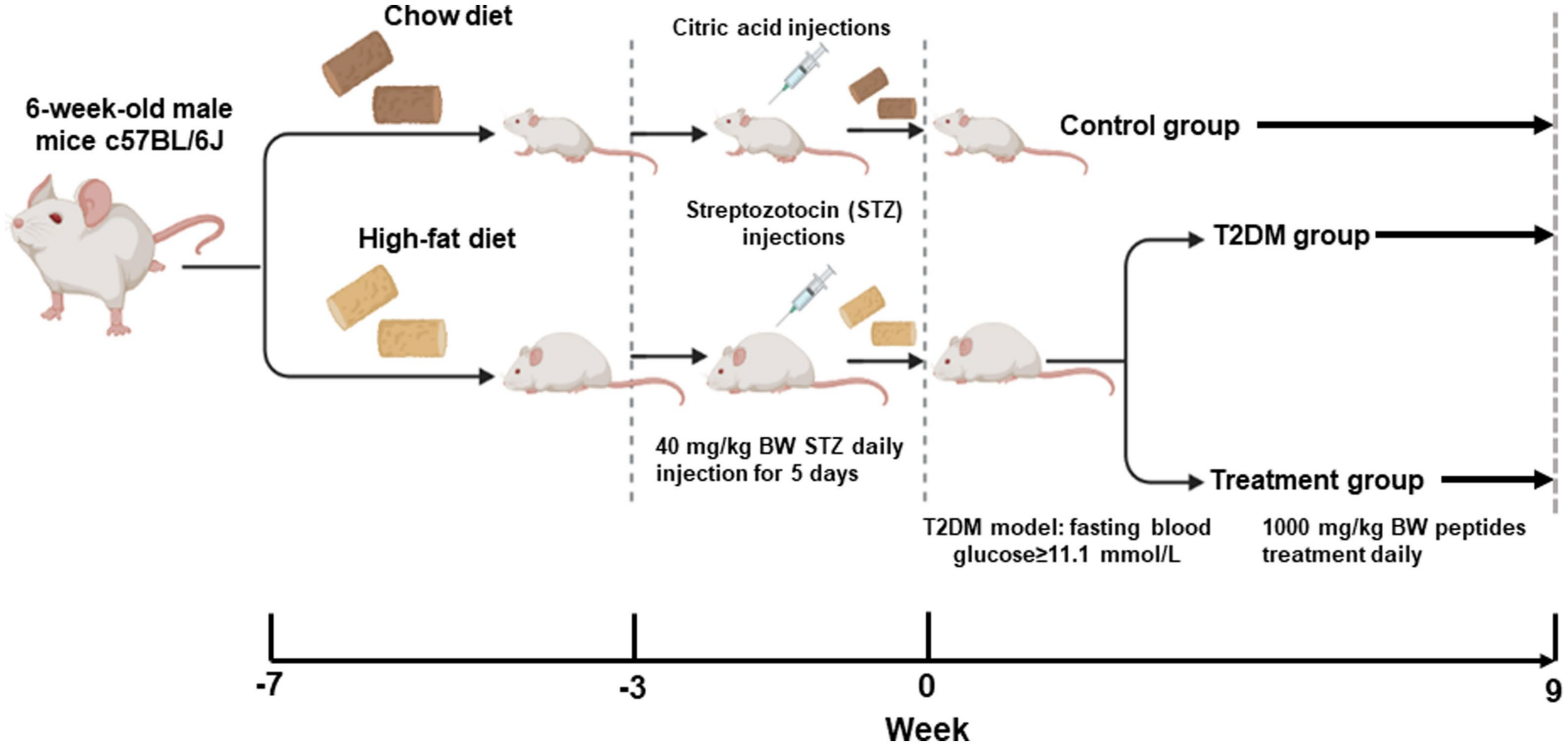
The timeline of the animal procedure.

The dosage and duration of the treatment were based on previous studies (16, 17). PPH which was produced via enzymatic hydrolysis was provided by Zhongshi Duqing (Shandong) Biotech Co., Ltd. (Heze, Shandong, China). The peptide composition accounts for 80% of the dried PPH powder. The peptide profile of PPH has been characterized in our previous study (11). PPH was dissolved in 0.5 mL of drinking water at a concentration of 1,000 mg/kg body weight and administered daily via oral gavage. The dosage used in this study could be translated to about 4 g/day to human based on the body surface area approach (18). Mice in the normal control and T2DM group received the same volume of drinking water daily. In week 0, week 5 and week 9 of the treatment, blood glucose of the animals after 12 h fasting was measured by blood glucose strip (Roche Diabetes Care, Inc., Basel, Switzerland) and glucose meter (Roche Diabetes Care). The procedure of the animal protocol is shown by Figure 1.

At the end of the treatment period, the oral glucose tolerance test (OGTT) was performed. Briefly, the animals were orally administered with 2 g/kg body weight glucose solution and blood glucose was measured by a glucose meter at 0.5 h, 1 h and 2 h post glucose gavage. At the end of the animal protocol, the animals were sacrificed and tissues were collected for the following assessments.

### 2.3 Liver histology

The liver tissues were fixed in 4% polyformaldehyde (Sinopharm Chemical Reagent Co., Ltd., Shanghai, China) for at least 24 h. The tissues were dehydrated by different concentrations of ethanol (Sinopharm Chemical Reagent Co., Ltd.) and embedded in paraffin. The tissues were sectioned at 4 μm. Afterwards, the sections were dewaxed in xylene (Sinopharm Chemical Reagent Co., Ltd.) and rehydrated by ethanol, which was followed by the staining with Haematoxylin and Eosin (H&E, Zhuhai BasO Biotechnology Co., Ltd., Zhuhai, Guangdong, China). At the end, the sections were dehydrated by ethanol and xylene prior to be covered and observed under the microscope (Shinjuku, Tokyo, Japan).

### 2.4 Serum assay

Blood samples were collected at the end point, followed by centrifugation under 1,000 × g for 15 min at 4°C and stored under −80°C. Serum levels of insulin, TNF-α, IL-6 and IL-1β were measured by the corresponding ELISA kit, which was purchase from Beyotime Biotechnology (Shanghai, China).

### 2.5 Western blotting

The liver tissue was homogenized in the RIPA buffer with protease and phosphatase inhibitors (GenStar, Beijing, China) and centrifuged at 4°C, 12,000 rpm for 10 min. The protein concentration was measured by the BCA assay (Thermo Fisher Scientific, Waltham, MA, United States). The protein sample (100 μg) from each animal was loaded to the stacking gel of SDS-PAGE and then separated by a 9% separating gel. The protein samples were then transferred into a PVDF membrane. Followed by the blocking using 5% defatted milk in TBST, the sample was incubated with specific primary antibody for overnight. GAPDH was used as the internal control. The antibody against Foxo1, p-Foxo1, CREB, p-CREB HO1, pp65, p65, p-Akt, Akt, p-IRS1, IRS1 and GAPDH were purchased from Cell Signaling Biotechnology (Danvers, MA, United States). The antibodies against AT1, ACE2 and MasR were purchased from Abcam (Cambridge, United Kingdom). The antibody against ACE was purchased from Proteintech Group, Inc. (Rosemont, IL, United States). The anti-rabbit or anti-mouse antibody, which was used as the secondary antibody, was purchased from Santa Cruz Biotechnology (Dallas, Texas, United States). The protein band was exposed by ECL detection reagent and detected by the Tanon chemiluminescent imaging system (Tanon, Shanghai, China). The density of the band was analyzed using the Image J software.^1^ Results were presented as the ratio of the phosphorylated protein and the total protein, or the ratio of the target protein and GAPDH.

### 2.6 qRT-PCR

Total RNA of the liver tissue was extracted using the TRIZOL reagent and cDNA was synthesized using the TAKARA PrimeScript^™^ RT reagent kit (Takara Bio, Kusatsu, Shiga, Japan). The SYBR green fast mix (Takara Bio) was used for amplification in a real-time PCR System (CFX Connect, Bio-Rad, Hercules, California, United States) with the specific primer (Table 2). GAPDH was used as the house-keeping gene. The relative mRNA level was calculated using the ΔCt method as follows: ΔCt = Ct_target_ − Ct_GAPDH_; ΔΔCt = ΔCt_treatment_ − ΔCt_control_; relative mRNA level = 2^−ΔΔCt^.

**Table 2:** The sequence of primers.

mRNA	Forward	Reverse
G6PC	5′-CATTGTGGCTTCCTTGGTCC-3′	5′-GGCAGTATGGGATAAGACTG-3′
TNF-α	5′ -GAGAAAGTCAACCTCCTCTCTG-3′	5′-GAAGACTCCTCCCAGGTATATG-3′
IL-6	5′-CCACTCCCAACAGACCTGTC-3′	5′-GGTACTCCAGAAGACCAGAGG-3′
IL-1β	5′-TGTTCTTTGAAGTTGACGGACCC-3′	5′-TCATCTCGGAGCCTGTAGTGC-3′
GADPH	5′-CACCCCATTTGATGTTAGTG-3′	5′-CCATTTGCAGTGGCAAAG-3′

### 2.7 Statistical analyses

Data were presented as mean ± SD from at least 3 animals of each group. The one-way analysis of variance (ANOVA) with the Tukey’s post-hoc test was used for the determination of statistical significance using PRISM 8 statistical software (GraphPad Software, San Diego, CA, United States). *p* < 0.05 was considered to be statistically significant.

## 3 Results

### 3.1 PPH reduced fasting blood glucose and improved glucose tolerance In T2DM mice

**Figure 2:**
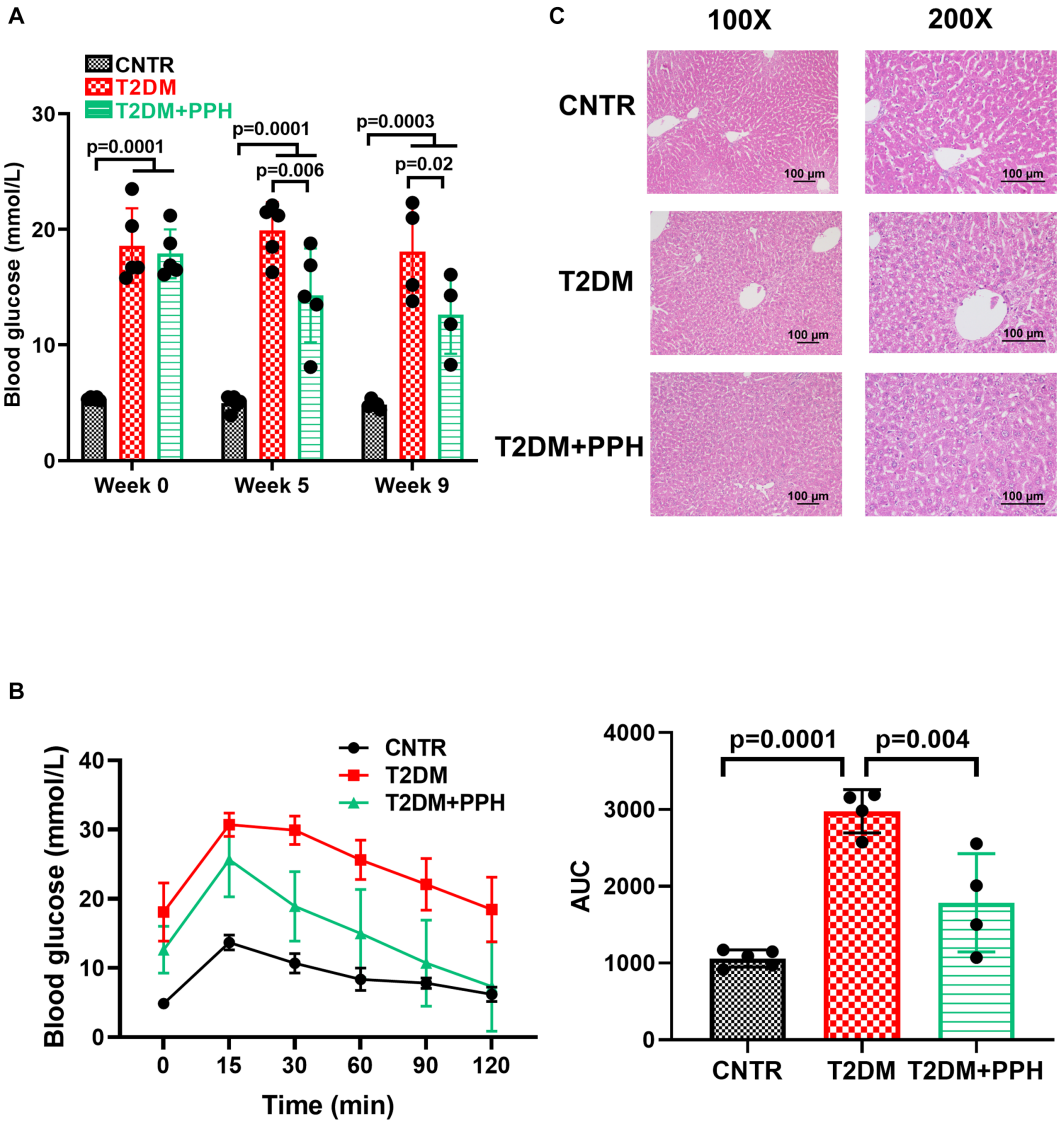
PPH reduced fasting blood glucose and improved glucose tolerance in T2DM mice. The T2DM mice induced by the high-fat-diet were orally administrated with 1,000 mg/kg body weight to the treatment group. **(A)** In week 0, week 5, and week 9, blood glucose of the animals after 12 h fasting was measured. **(B)** At the end of the treatment period, the oral glucose tolerance test (OGTT) was performed. The animals were orally administered with 2 g/kg body weight glucose solution and blood glucose was measured by a glucose meter at 0.5 h, 1 h and 2 h post glucose gavage. **(C)** H&E staining was used for the liver histology. Results are presented as mean ± SD from 4–5 animals of each group.

As expected, high-fat-diet and low dose of STZ injections significantly increased the fasting blood glucose (Figure 2A), suggesting that the T2DM mouse model was established successfully in our study. The fasting blood glucose of the T2DM mice was reduced significantly by the PPH treatment for 5 weeks (Figure 2A, T2DM: 19.92 mmol/L versus T2DM + PPH: 14.3 mmol/L). The mean blood glucose of the PPH treatment group was further reduced to 12.63 mmol/L (29.6% reduction) after 9 weeks of treatment. The above results suggested that PPH treatment could reduce fasting blood glucose in T2DM mice. In addition to the fasting blood glucose, the impaired glucose tolerance in the T2DM mice could be improved by the PPH treatment (Figure 2B). Moreover, results from the H&E staining showed a significant lipid accumulation in the liver of T2DM mice (Figure 2C), while such an effect could be normalized by the PPH treatment. Although the level of hepatic triglycerides was measured, non-significant change was observed by the PPH treatment (data not shown).

### 3.2 PPH improved hepatic gluconeogenesis and ameliorated inflammation

**Figure 3:**
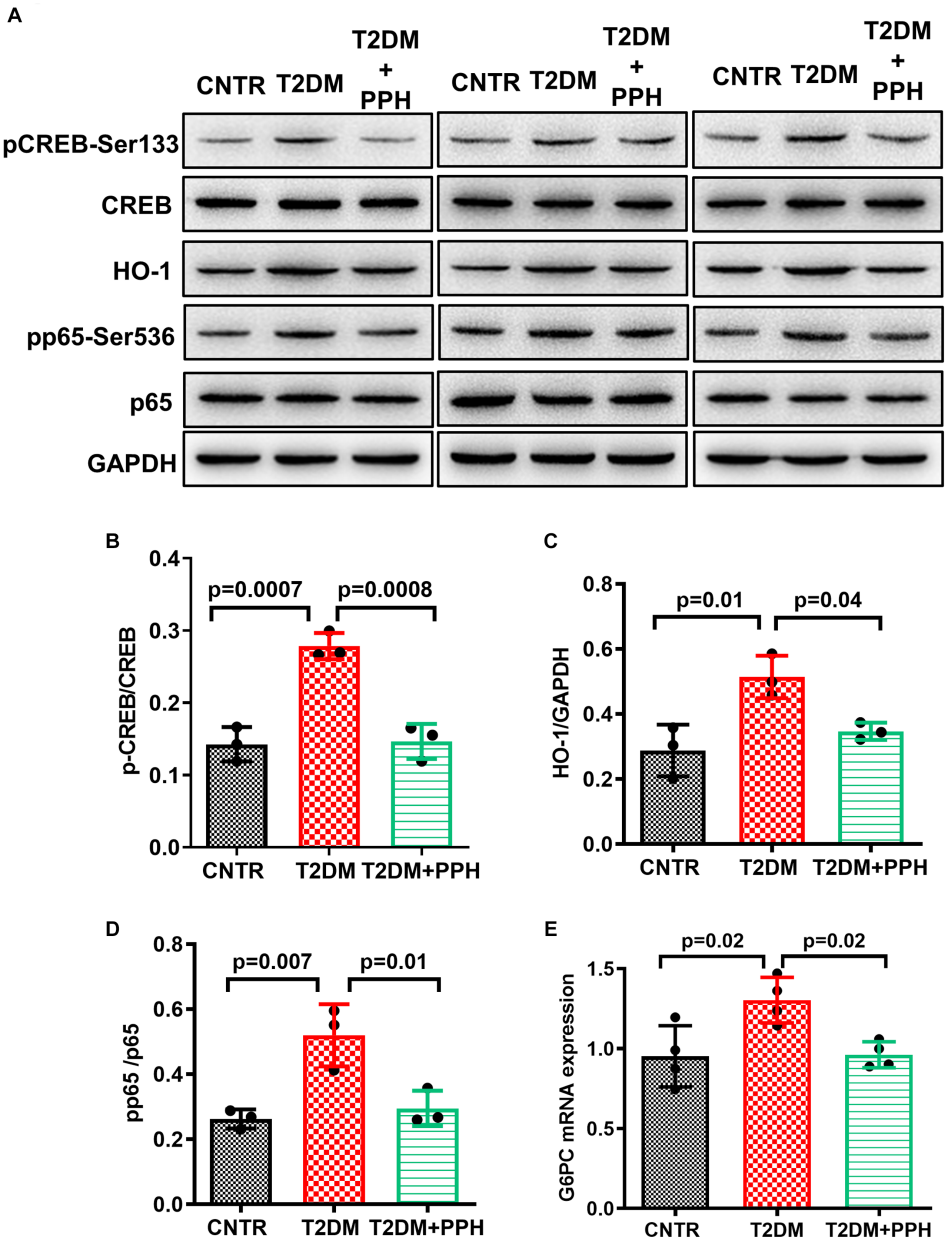
PPH improved hepatic gluconeogenesis in T2DM mice. The liver tissues were collected at the end of the animal protocol. **(A)** Total proteins were extracted and immune-blotted with specific antibody. **(B–E)** The protein bands of pCREB-Ser133 and pp65-Ser536 were quantified and normalized to the corresponding total form. The protein band of HO1 was quantified and normalized to GAPDH. Results are presented as mean ± SD from 3 animals of each group.

Since PPH treatment reduced fasting blood glucose in T2DM mice, we then examined the hepatic gluconeogenic pathway. The phosphorylation of CREB in the liver was significantly improved in the T2DM mice, which was normalized by the PPH treatment (47.5% reduction as compared with the T2DM group, Figures 3A,B). In addition, the mRNA level of G6PC, which is a gluconeogenic gene, was normalized by the PPH treatment as well (26.2% reduction as compared with the T2DM group, Figure 3E). These results suggested that PPH treatment could modulate the dysregulated gluconeogenesis in the T2DM mice.

We have previously found that the activation of CREB signaling increased hepatic inflammation via HO1 and NF-kB signaling (19). It was found in the present study that the protein level of HO1 was enhanced in the T2DM mouse liver, but it could be alleviated by the PPH treatment (32.5% reduction as compared with the T2DM group, Figures 3A,C). The change in the phosphorylation of p65 (43.3% reduction as compared with the T2DM group, Figures 3A,D) was in the same pattern as HO1. The above results indicated the potential of PPH in mitigating hepatic inflammation in T2DM mice.

**Figure 4:**
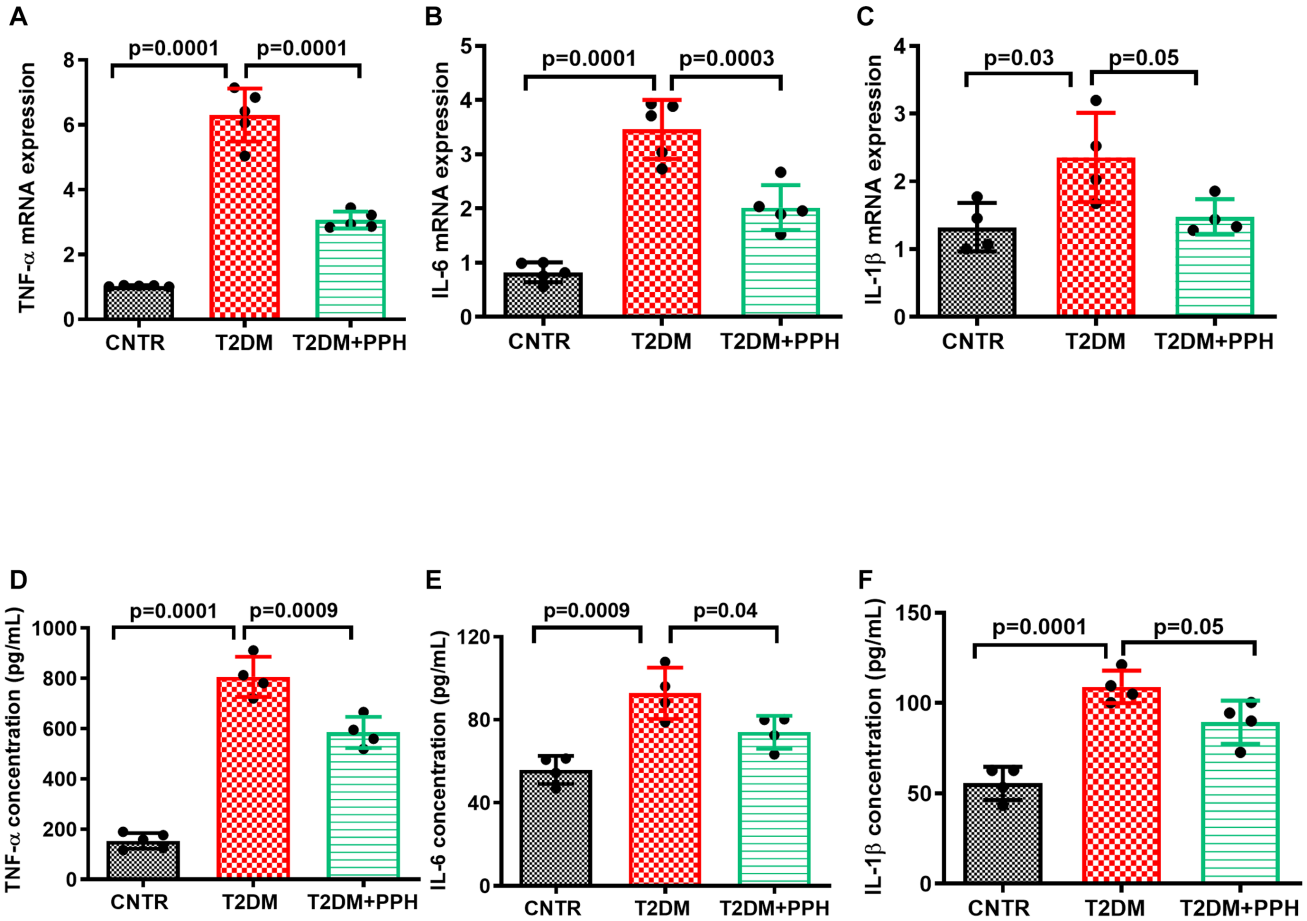
PPH ameliorated inflammation in T2DM mice. The liver tissues and blood samples were collected at the end of the animal protocol. **(A–C)** Total RNA of the liver tissue was extracted and cDNA was synthesized. The mRNA levels of TNF-α, IL-6 and IL-1β were detected by qRT-PCR. **(D–F)** The concentrations of TNF-α, IL-6 and IL-1β in the serum were measured by the corresponding ELISA kit. Results are presented as mean ± SD from 4–5 animals of each group.

In addition to the pro-inflammatory signaling in the liver, mRNA levels of pro-inflammatory markers including TNF-α, IL-6 and IL-1β (Figures 4A–C) were significantly increased in the T2DM mice liver, which were reduced by the PPH treatment by 51.4%, 41.7%, and 37.2%, respectively. The concentration of these molecules in the serum were also decreased by the PPH treatment as compared with the T2DM model (TNF-α: 27.5% reduction, IL-6: 20.2% reduction, IL-1β: 18.0% reduction, Figures 4D–F).

### 3.3 PPH improved hepatic insulin sensitivity

**Figure 5:**
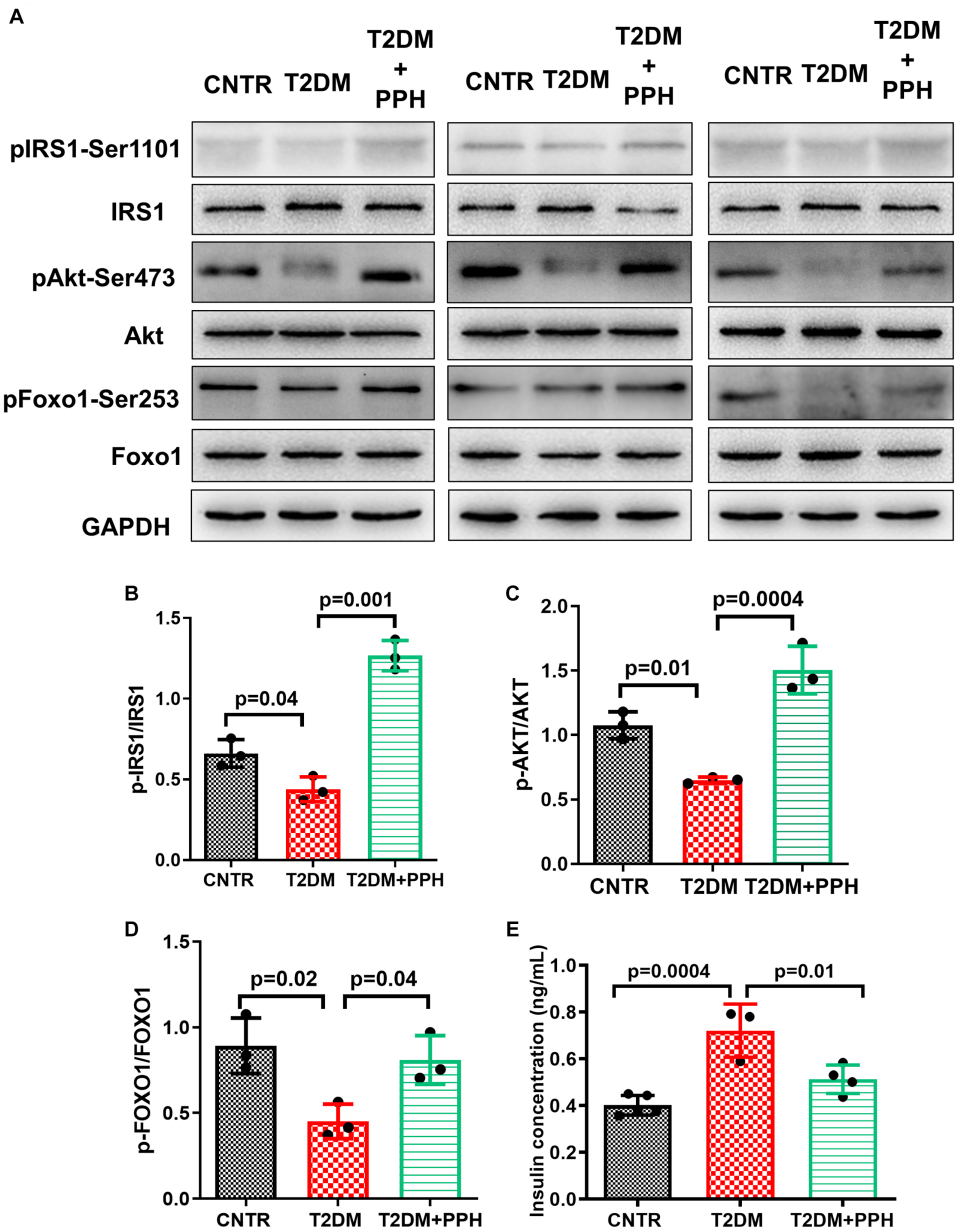
PPH improved hepatic insulin sensitivity in T2DM mice. The liver tissues were collected at the end of the animal protocol. **(A)** Total proteins were extracted and immune-blotted with specific antibody. **(B–D)** The protein bands of pIRS1-Ser1101, pAkt-Ser473 and pFOXO1-Ser253 were quantified and normalized to the corresponding total form. **(E)** The insulin concentration in the serum was measured by an ELISA it. Results are presented as mean ± SD from 3–5 animals of each group.

The T2DM mouse model established by high-fat-diet and low dose STZ showed an impaired hepatic insulin sensitivity (Figure 5). In addition to the hepatic gluconeogenic signaling, the insulin signaling was also activated by the PPH treatment, which was evident by the enhanced phosphorylation of IRS-1 (188.9% increase as compared with the T2DM group, Figures 5A, 4B), Akt (Figures 5A,C) and Foxo1 (Figures 5A,D). Moreover, the serum insulin level (Figure 5E) was normalized by the PPH treatment (28.8% reduction as compared with the T2DM group), which further indicated that the insulin sensitivity of T2DM mouse could be improved by the PPH treatment.

### 3.4 PPH modulated the components of the hepatic renin angiotensin system

**Figure 6:**
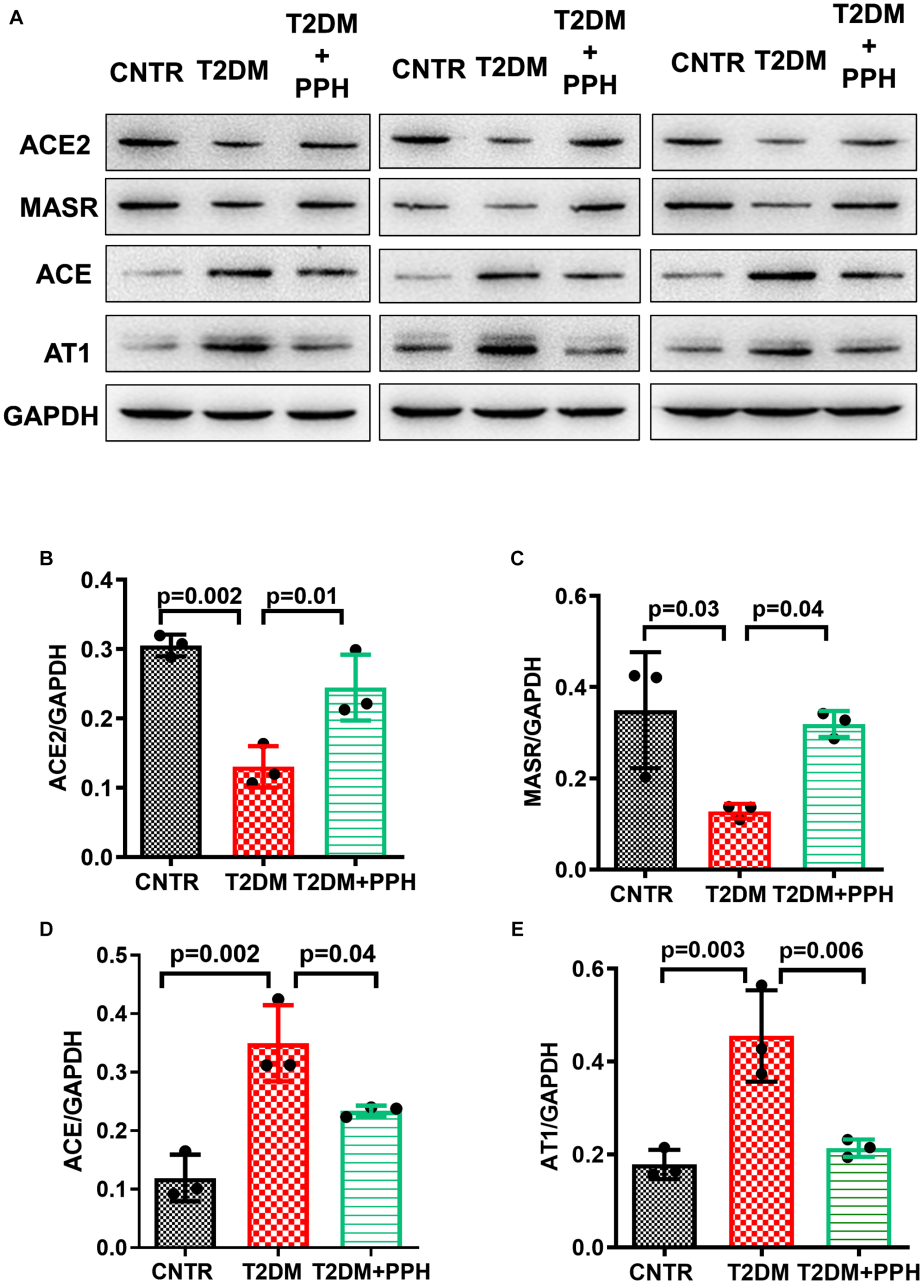
PPH modulated the components of the hepatic RAS. The liver tissues were collected at the end of the animal protocol. **(A)** Total proteins were extracted and immune-blotted with specific antibody. **(B–E)** The protein bands of ACE2, MASR ACE and AT1 were quantified and normalized to GAPDH. Results are presented as mean ± SD from 3 animals of each group.

Since we previously reported the activity of PPH in upregulating ACE2 expression in vascular smooth muscle cells (20), we then explored whether PPH treatment regulated the expressions of renin angiotensin system (RAS) components in the liver of T2DM mice. The protein levels of ACE2 and MasR were significantly reduced in the liver of T2DM mice (57.3% reduction and 63.4% reduction, respectively, as compared with the CNTR group, Figures 6A–C), while the protein levels of AT1 and ACE were increased (155.1% increase and 191.7% increase, respectively, as compared with the CNTR group, Figures 6A,D,E). Notably, the treatment of PPH showed a counter-balancing effect in regulating these RAS components (Figure 6).

## 4 Discussion

In the past years, the anti-diabetic activity of legume-derived bioactive peptides have been examined. However, the majority of these studies showed the hypoglycemic effect of soybean peptides, the knowledge of pea peptides is still limited (8). In addition, the *in vivo* molecular mechanisms underlying the glycemic controlling effect of the legume-derived peptides need to be elucidated. Herein, we investigated the hypoglycemic effect of PPH in a T2DM mouse model in the present study and key findings were as follows: (1). PPH reduced fasting blood glucose and improved glucose tolerance in the HFD and low-dose STZ-induced T2DM mouse model; (2). The PPH treatment suppressed the hepatic gluconeogenic signaling but activated the hepatic insulin signaling in the T2DM mice; (3). PPH mitigated inflammation in the T2DM mice; (4). The PPH treatment modulated the RAS component of liver in the T2DM mice.

Under the fasting status, glucagon degrades macromolecules and activates hepatic gluconeogenesis (21). The fasting blood glucose of the T2DM mice could be significantly reduced by the PPH treatment, which indicated that the abnormal gluconeogenesis was repaired by the treatment. This finding was also demonstrated by the reduced phosphorylation level of CREB-Ser133, a key molecule in the gluconeogenic signaling, as well as the reduced mRNA level of G6PC, a gluconeogenic gene. Of note, our previous study found that the PPH treatment could suppress glucose production via inhibiting gluconeogenic signaling in the hepatic AML-12 cells (11), results from the present study further provided *in vivo* demonstration of this finding. Although there have been a number of studies reporting the *in vivo* hypoglycemic activity of food protein-derived bioactive peptides (13, 22–24), the effect of peptides on gluconeogenesis was rarely discussed. The finding from this study showed a novel mechanism of bioactive peptides in reducing blood glucose, which is through the regulation of hepatic gluconeogenic pathway.

Notably, the inflammation of the T2DM mice was ameliorated by the PPH treatment, which was demonstrated by the decreased levels of pro-inflammatory molecules in both of the hepatic tissues and circulation. Such an effect was associated with the reduced protein levels of HO1 and the phosphorylated p65/NF-κB. We previously reported that upregulation of HO1, which is in the down-stream of the gluconeogenic signaling, could activate the p65/NF-κB signaling and enhance inflammation via producing excessive ferrous iron (19). Since the iron overload is related to the elevated blood glucose (25, 26), it would be of interest to further investigate the regulatory role of PPH in iron homeostasis.

The effect of bioactive peptides on activating the insulin signaling has been documented in soybean, bovine milk and the egg white-derived peptides (27). In this study, the activation of insulin signaling by the pea protein-derived peptides was evident in the liver of T2DM mice. Such activation could further contribute to improving insulin sensitivity, which was demonstrated by the improvement of the glucose tolerance as well as the reduced insulin level in the serum. Interestingly, our previous cellular study showed that the treatment of PPH suppressed the glucose production in hepatic cells via the gluconeogenic signaling but not the insulin signaling (11). It is suggested to further examine the contribution of the gluconeogenic signaling and the insulin signaling in mediating the hypoglycemic effect of PPH.

In addition, the dysregulated blood glucose is concomitant with the abnormality of lipid metabolism (28, 29). The dual effects of bioactive peptides in regulating glucose and lipid metabolism have been investigated. It has been reported that the intervention of marine collagen peptides could modulate glucose and lipid metabolism in patients with T2DM (30). It was also showed that the casein hydrolysate could reduce the levels of blood glucose and triglycerides in overweight or obese subjects (31). More recently, an egg white-derived peptide was reported with the potential to ameliorate non-alcoholic fatty liver in insulin resistant mice (32). Of note, it was found in this study that lipid deposition occurred in the liver of T2DM mice, while the treatment of PPH showed a counter-balancing effect. Hereby, further study of PPH could consider investigating the glycemic controlling effect with the cross-talk of lipid metabolism, which may provide new opportunity for the application of PPH in the management of nutrition related chronic diseases. Moreover, since the liver is a central organ in lipogenesis, gluconeogenesis and cholesterol metabolism (33), the abnormality of these biological process as well as their connections have been widely discussed, in which, the role of proinflammatory molecules also involved. The lipid deposition in the liver could decrease hepatic blood flow, which then promote the formation of reactive oxygen species and hepatic inflammation (34). On the other hand, proinflammatory markers such as IL-6 and TNF-α play a role in glucose metabolism (35). Given the multiple effects of PPH on blood glucose, inflammatory markers, and hepatic lipid deposition in the T2DM mice, it would be of interest to further investigate the cross-talks of these effects.

The RAS plays a major role in regulating blood pressure, while emerging evidence suggested that dysregulated RAS might be associated with metabolic disorders, such as hyperglycemia (36). In particular, the hepatic RAS was implicated to affect the hepatic glucose production (37). In the RAS, the ACE/AT1 axis exerts detrimental effects, while, the ACE2/MasR axis plays a counter-balancing role (38). Besides, the regulatory effects of pea protein-derived peptides on the RAS components have been documented (39). Notably, the present study found that the treatment of PPH up-regulated ACE2 and MasR expressions but down-regulates ACE and AT1 expressions in the liver of T2DM mouse. In our previous study, we identified peptides from pea proteins that could up-regulate ACE2 protein expression in vascular smooth muscle cells (20). In addition, it was reported that LRW, a tripeptide which is also characterized from pea protein, improved ACE2 protein expression in vascular cells (40). Results from this study further indicate the potential of pea protein-derived peptides in modulating the RAS components. The role of RAS in mediating the effects of PPH is to be determined in future studies.

## 5 Conclusion

In conclusion, the hypoglycemic effect of PPH in T2DM mice was demonstrated in the present study. The underlying molecular mechanisms include suppression of gluconeogenic signaling, activation of insulin signaling, and modulation of the RAS. Findings from this study indicate the potential of PPH to be further developed into functional foods or nutraceuticals. However, clinical effects of PPH in regulating glucose metabolism should be evident by the follow-up studies.

## Data availability statement

The original contributions presented in the study are included in the article/supplementary material, further inquiries can be directed to the corresponding author.

## Ethics statement

The animal study was approved by Animal Care and Use Committee of the Southeast University (Approval #20210221003). The study was conducted in accordance with the local legislation and institutional requirements.

## Author contributions

WL: Conceptualization, Investigation, Writing – original draft. XC: Data curation, Investigation, Writing – original draft. HX: Methodology, Writing – review & editing. SW: Methodology, Writing – review & editing. LC: Methodology, Writing – review & editing. GS: Conceptualization, Project administration, Writing – review & editing.

## Footnotes

^1^ https://imagej.nih.gov/ij/
